# Comparison of Neuropsychological Characteristics in Children and Adolescents with Autism Spectrum Disorder and Tic Disorder

**DOI:** 10.1192/j.eurpsy.2025.1157

**Published:** 2025-08-26

**Authors:** S. Lee, S. Y. Lee, B.-N. Kim

**Affiliations:** 1 Seoul National University Hospital, Seoul, Korea, Republic Of

## Abstract

**Introduction:**

Individuals with autism spectrum disorder (ASD) show notable intellectual impairment compared to other neurodevelopmental disorders, as seen in intelligence and neuropsychological tests, including attention assessments. Given the strong link between intellectual and neuropsychological performance, it is essential to determine if low neuropsychological scores stem from general intellectual limitations or specific domain vulnerabilities.

**Objectives:**

To assess specific neuropsychological vulnerabilities in ASD, we will administer intelligence and neuropsychological tests to both ASD and Tic disorder groups, examining group differences after controlling for FIQ. Within the ASD group, we will further analyze performance variations by symptom severity and explore links between test results and specific ASD symptoms.

**Methods:**

We recruited children aged 5-16 with no comorbidities into Tic (n=75) and ASD (n=97) groups, administering the Wechsler Intelligence Scale and neuropsychological tests (ATA, CCTT, WCST, Stroop). Using SPSS 29, we performed an ANCOVA with FIQ as a covariate to examine group differences. In the ASD group, ADOS-2 scores further distinguished autism spectrum and autism groups, and correlations were analyzed between neuropsychological scores and ADOS-2 Social, RRB, and Total scores.

**Results:**

The ASD group had a significantly lower FIQ than the Tic group (t(66) = 2.54, p = .013) and showed poorer WCST performance with fewer correct responses and more errors, even after controlling for FIQ. In the ATA, the ASD group’s response time was significantly faster, with higher false alarms, indicating hasty judgments. The Stroop color-word task showed poorer ASD performance, while the CCTT interA_rate was higher in the Tic group. No significant differences were found between the autism spectrum and autism groups on neuropsychological tests, except intelligence. ATA scores correlated significantly with ADOS-2 social, RBB, and total scores, whereas WCST scores correlated only with RBB.

**Conclusions:**

This study’s WCST, ATA, and Stroop test results indicate that the ASD group has deficits in cognitive flexibility and inhibitory control, independent of FIQ. The absence of significant differences between autism and spectrum groups suggests these traits are inherent to the autism spectrum, regardless of symptom severity. Unexpectedly, the CCTT interA_rate was high, likely due to a floor effect given the ASD group’s longer CCTT 1 completion time. ATA scores correlated with overall autism symptoms, while WCST scores correlated only with RBB, linking social interaction difficulties to attentional control and repetitive behaviors to cognitive rigidity and insistence on sameness.

**Disclosure of Interest:**

S. Lee Conflict with: Financial Support for Research from: This research was supported by the National IT Industry Promotion Agency (NIPA), an agency under the MSIT and with the support of the Daegu Digital Innovation Promotion Agency (DIP), the organization under the Daegu Metropolitan Government, and co-funded by the National Research Foundation (NRF) funded by the Korean Government (MSIT) (RS-2024-00397737)., S. Y. Lee: None Declared, B.-N. Kim: None Declared

